# Changes in the medical treatment status of Japanese outpatients during the coronavirus disease 2019 pandemic

**DOI:** 10.1002/jgf2.432

**Published:** 2021-03-16

**Authors:** Takeshi Takakubo, Yuko Odagiri, Masaki Machida, Tomoko Takamiya, Noritoshi Fukushima, Hiroyuki Kikuchi, Shiho Amagasa, Itaru Nakamura, Hidehiro Watanabe, Shigeru Inoue

**Affiliations:** ^1^ Department of Preventive Medicine and Public Health Tokyo Medical University Tokyo Japan; ^2^ Department of Infection Prevention and Control Tokyo Medical University Hospital Tokyo Japan

**Keywords:** COVID‐19, decrease in medical visit frequency, fear of infection at medical facilities, inability to take regular medications, utilization of telephone/online medical care

## Abstract

**Background:**

The coronavirus disease 2019 (COVID‐19) has a tremendous influence in general public's behaviors; however, changes in the status of regularly scheduled outpatient visits in Japan during COVID‐19 pandemic are still unknown.

**Methods:**

This cross‐sectional study was conducted in May 2020. Participants were recruited by an Internet‐based survey company. A total of 659 patients (54% male, average age 60 ± 14 years) who had regularly scheduled outpatient visits prior to the onset of COVID‐19 were enrolled. Participants answered four questions (“decrease in medical visit frequency,” “inability to take regular medication,” “deterioration of a chronic disease,” and “utilization of telephone/online medical care”) and stated whether they had a fear of acquiring infection at a medical facility. The associations between answers, fear of infection, and socio‐demographic factors were examined.

**Results:**

Among the participants, 37.8% had decreased their medical visits, 6.8% were unable to take regular medications, 5.6% experienced a deterioration of chronic disease, and 9.1% utilized telephone/online medical care. Fear of being infected by COVID‐19 at medical facilities was strongly associated with a reduced frequency of medical visits and lack of regular medications even after adjusting for socio‐demographic factors and current medical histories.

**Conclusions:**

During the first wave of COVID‐19, approximately 40% of participants reduced their frequency of medical visits. It is important to continue implementing thorough infection control measures at facilities and educating the public the importance of keeping chronic diseases in good condition, as well as promoting telephone/online medical care.

## Graphical abstract

We conducted an Internet survey of 2,400 Japanese men and women in May 2020 and analyzed changes in the medical visit behavior/status of 659 people who had regularly visited to medical institutions before the coronavirus disease 2019 pandemic. Among the participants, 37.8% had decreased their medical visits, 6.8% were unable to take regular medications, 5.6% experienced a deterioration of chronic disease, and 9.1% utilized telephone/online medical care.

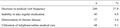

## 1 INTRODUCTION

The coronavirus disease 2019 (COVID‐19) epidemic has become a global challenge. Because of the nationwide spread of COVID‐19 infections and the increasing number of deaths,^1^, ^2^ a state of emergency was declared in April 2020 by the Japanese Ministry of Health, Labor and Welfare (JMHLW). As COVID‐19 spreads, there have been clusters of COVID‐19 reported at medical facilities,^3^ and patients have refrained from visiting them. Under the current circumstances, even though many citizens practice infection prevention measures, COVID‐19 cases are not decreasing.

Studies have shown that COVID‐19 is more serious in the elderly and those with hypertension, diabetes, heart disease, chronic respiratory disease, and cancer.^4^, ^5^, ^6^, ^7^, ^8^, ^9^ Therefore, it is particularly important that these patients keep their diseases under control to prevent death. During the COVID‐19 pandemic, the number of outpatient visits at many medical facilities decreased, according to aggregated data from outpatient billing receipts.^10^ Changes in the medical treatment status of Japanese outpatients (decrease in visit frequency, inability to take regular medications, and deterioration of chronic disease) and socio‐demographic factors that influenced outpatient status are not well understood. Therefore, this study was conducted to examine changes in the treatment behavior and health status of patients undergoing regular outpatient visits and to identify the factors that influenced those behaviors during the COVID‐19 pandemic.

## 2 MATERIALS AND METHODS

### 2.1 Study design and subjects

This study was a cross‐sectional study utilizing the data obtained for some studies,^11^, ^12^, ^13^ which has been conducted to examine the influence of the COVID‐19 on general Japanese population. Participants were recruited from a Japanese Internet‐based survey company that has approximately 1.12 million registrants as of January 2020. We collected data from 2400 people aged 20‐79 years (sampling by gender and 10‐year age group; 12 groups, n = 200 in each group) who were living in the Tokyo metropolitan area. The company randomly chosen potential respondents from the registrants. The number of potential respondents in each stratified sample group was determined by dividing 200 by the response rate for the corresponding socio‐demographic group. The response rate was then computed based of the results of past surveys conducted by the research company. The company sent an email on February 25, 2020, inviting registrants to participate in the survey (n = 8156).

Survey questions were sent via a URL in an email. Once 200 people in each group responded, we closed the group. The first survey was completed on February 27. Participants were given points equivalent to 50 Japanese yen (JPY) per response. The third survey, which included items regarding the status of outpatient visits, was conducted between May 12 and 17 with 2400 subjects. This third survey was conducted after the state of emergency, that had been declared in seven prefectures on April 7 and was expanded nationwide on April 16,^14^ was lifted in some prefectures.

In the third survey, we asked participants whether they had had regular outpatient visits before the onset of COVID‐19 (as of fall 2019). Participants who answered “I had regular visits to the hospital outpatient department” or “I had regular visits to the clinic” were included in the study. We excluded respondents who only selected pollinosis (hay fever) from the list of potential current medical conditions, as pollinosis is often treated with over‐the‐counter medication and does not necessarily lead to an outpatient visit. Respondents who were missing socio‐demographic information or whose answers were of suspect accuracy or honesty were also excluded.

### 2.2 Measurement

Changes in outpatient visit status: We asked participants about the status of their outpatient visits: a decrease in medical visit frequency, an inability to take regular medications, a deterioration of a chronic illness, and utilization of telephone/online medical care (yes/no format).

Fear of being infected at medical facilities: We asked participants if they were afraid of being infected with COVID‐19 at medical facilities (yes / no format).

Assessment of socio‐demographic attributes and lifestyle‐related factors: Gender, age, residential area (Tokyo Metropolis / Tokyo suburbs [Saitama Prefecture / Chiba Prefecture / Kanagawa Prefecture] / Northern Kanto [Gunma Prefecture / Tochigi Prefecture / Ibaraki Prefecture]), marital status (single, divorced, or widowed), living arrangement (alone or with others), education (university graduate or above/high school or below), working status (yes / no format), and annual household income (<5 million JPY / ≥5 million JPY) provided by the research company were used.

Smoking status (smoking / nonsmoking), alcohol consumption (≥3 days per week / <2 days per week), and exercise habits (≥30 minutes of light sweating exercise twice a week / none) were reported. Participants also rated their health (very good, good, not very good, and not good) and reported their current illnesses (hypertension, diabetes, heart disease, stroke, respiratory disease, and cancer).

### 2.3 Statistical analysis

The prevalence of those who answered yes to 4 medical treatment status, that is, “decrease in medical visit frequency,” “inability to take regular medication,” “deterioration of chronic illness,” and “utilization of telephone/online medical care,” was calculated for each socio‐demographic attribute and compared using the chi‐square test.

Multivariable logistic regression analysis was performed to clarify the factors independently related to the medical treatment situation. The dependent variables were four measurement of medical treatment status. The independent variables of model 1 were gender, age, residential area, marital status, living arrangement, education, working status, annual household income, lifestyle (smoking, alcohol consumption, and exercise habits), self‐rated health, type of medical facility, and fear of being infected at medical facilities. In model 2, in addition to the independent variables in model 1, the presence or absence of a current illness (hypertension, diabetes, heart disease, stroke, respiratory disease, or cancer) (yes/no format) was added. In model 3, in addition to the independent variables discussed in model 2, decrease in medical visit frequency was included as an independent variable.

Statistical analyses were performed using IBM SPSS Statistics for MAC, version 26 (IBM Japan). The significance level was set at *P* < .05.

## 3 RESULTS

**FIGURE 1:**
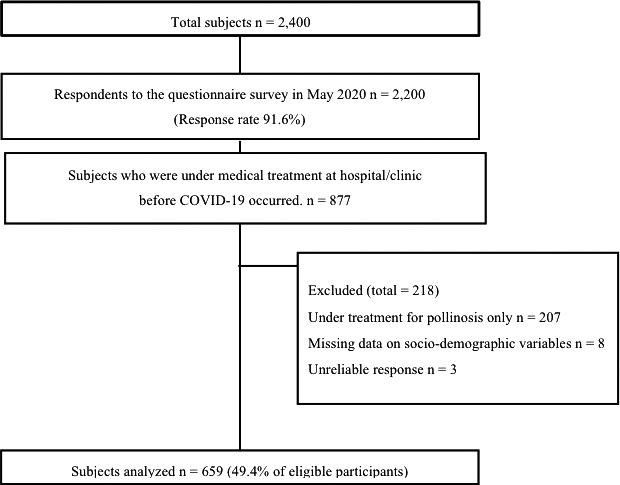
Participant flow

A total of 877 people met the inclusion criteria of “visited hospitals or clinics” before the fall of 2019. Respondents were excluded as follows: only reporting pollinosis for their current medical history (n = 207), lacking basic information (n = 8), and suspect credibility (n = 3) (specifically, 3 men in their 20s who answered that they had a history of ≥4 diseases when asked about their current medical history; the validity of the data was judged to be low by two researchers independently). As a result, 659 people (75.1%) were included in the study (Figure 1).

Table 1 shows the socio‐demographic characteristics of the participants. In total, 54.2% of the participants were male. Sixty‐one percentage of the participants were 60‐79 years, 33.8% lived in the Tokyo Metropolis. The location of visits was divided fairly equally between hospitals (55.8%) and clinics (44.2%). Three‐quarters (75.3%) of the respondents answered that they were afraid of being infected with COVID‐19 at a medical facility.

**TABLE 1:** Participant characteristics (n = 659)

	n	%
Gender		
Male	357	54.2
Female	302	45.8
Age (years)		
20‐39	82	12.4
40‐59	174	26.4
60‐79	403	61.1
Residential area		
Tokyo Metropolis	223	33.8
Tokyo suburbs^a^	360	54.6
Northern Kanto^b^	76	11.5
Marital status		
Married	452	68.6
Single, divorced, widowed	207	31.4
Living arrangement		
Living alone	113	17.1
Living with others	546	82.9
Education		
University graduate or above	350	53.1
Less than junior college graduate	309	46.9
Working status		
Yes	336	51.0
No	323	49.0
Annual household income		
Less than 5 million JPY	329	49.9
More than 5 million JPY	330	50.1
Smoking status		
Smoker	104	15.8
Nonsmoker or ex‐smoker	555	84.2
Alcohol consumption		
More than 3 d/wk	235	35.7
Less than 3 d/wk	424	64.3
Exercise habits^c^		
Yes	244	37.0
No	415	63.0
Self‐rated health		
Good, very good	526	79.8
Not very well, not good	133	20.1
Type of medical facilities participants were visiting		
Hospital	368	55.8
Clinic	291	44.2
Fear of infection at medical facilities		
Yes	496	75.3
No	163	24.7
Current medical histories		
Hypertension	324	49.2
Diabetes	104	15.8
Heart disease	53	8.00
Stroke	14	2.10
Respiratory disease	58	8.80
Cancer	36	5.50
Others / None of above	217	31.7

^a^ Tokyo suburbs include Saitama, Chiba, and Kanagawa prefecture.

^b^ Northern Kanto includes Gunma, Tochigi, and Ibaraki prefecture.

^c^ Exercise habits are defined as participating light sweating exercise regularly 3 times a week.

Treatment‐related behavior and status of all participants are shown in Table 2. A decrease in medical visit frequency was reported by 37.8%, but an inability to take regular medication was reported by only 6.8% of participants and deterioration of a chronic illness by 5.6%. Furthermore, 9.1% utilized telephone/online medical care.

**TABLE 2:** Treatment‐related behavior/status (n = 659)

	n	%
Decrease in medical visit frequency	249	37.8
Inability to take regular medication	45	6.8
Deterioration of chronic disease	37	5.6
Utilization of telephone/online medical care	60	9.1

The status of medical visits/behaviors by socio‐demographic factors is presented in Table 3.

**TABLE 3:** Prevalence of treatment‐related behaviors and status by socio‐demographic and lifestyle‐related factors

		Decrease in medical visit frequency			Inability to take regular medication			Deterioration of chronic disease			Utilization of telephone/online medical care		
		n	%	*P*‐value*	n	%	*P*‐value*	n	%	*P*‐value*	n	%	*P*‐value*
	(n = 659)	249	37.8		45	6.8		37	5.6		60	9.1	
Gender													
Female	302	135	44.7	<.001	20	6.6	.847	20	6.6	.301	30	9.9	.496
Male	357	114	31.9		25	7.0		17	4.8		30	8.4	
Age (years)													
20‐39	82	37	45.1	.218	13	15.9	.001	12	14.6	<.001	11	13.4	.017
40‐59	174	69	39.7		14	8.0		15	8.6		7	4.0	
60‐79	403	143	35.5		18	4.5		10	2.5		42	10.4	
Residential area													
Tokyo Metropolis	223	97	43.5	.035	16	7.2	.603	15	6.7	.202	26	11.7	.244
Tokyo suburbs^a^	360	131	36.4		22	6.1		21	5.8		29	8.1	
Northern Kanto^b^	76	21	27.6		7	9.2		1	1.3		5	6.6	
Marital status													
Married	452	177	39.2	.282	32	7.1	.706	22	4.9	.218	43	9.5	.590
Single, divorced, widowed	207	72	34.8		13	6.3		15	7.2		17	8.2	
Living arrangement													
Living alone	113	44	38.9	.781	6	5.3	.482	9	8.0	.233	12	10.6	.539
Living with others	546	205	37.5		39	7.1		28	5.1		48	8.8	
Education													
University graduate or above	350	131	37.4	.841	26	7.4	.516	18	5.1	.576	33	9.4	.758
Less than junior college graduate	309	118	38.2		19	6.1		19	6.1		27	8.7	
Working status													
Yes	336	125	37.2	.753	29	8.6	.061	24	7.1	.082	25	7.4	.130
No	323	124	38.4		16	5.0		13	4.0		35	10.8	
Annual household income													
Less than 5 million JPY	329	114	34.7	.098	20	6.1	.446	16	4.9	.403	31	9.4	.777
More than 5 million JPY	330	135	40.9		25	7.6		21	6.4		29	8.8	
Smoking status													
Smoker	104	36	34.6	.468	11	10.6	.099	9	8.7	.142	7	6.7	.359
Nonsmoker or ex‐smoker	555	213	38.4		34	6.1		28	5.0		53	9.5	
Alcohol consumption													
More than 3days / week	235	92	39.1	.591	17	7.2	.759	8	3.4	.067	24	10.2	.462
Less than 3days / week	424	157	37.0		28	6.6		29	6.8		36	8.5	
Exercise habits^c^													
Yes	244	96	39.3	.527	16	6.6	.832	13	5.3	.806	35	14.3	<.001
No	415	153	36.9		29	7.0		24	5.8		25	6.0	
Self‐rated health													
Very good, good	526	191	36.3	.121	37	7.0	.677	17	3.2	<.001	50	9.5	.477
Somehow no good, no good	133	58	43.6		8	6.0		20	15.0		10	7.5	
Type of medical facilities participants were visiting													
Hospital	368	129	35.1	.104	27	7.3	.561	27	7.3	.031	39	10.6	.134
Clinic	291	120	41.2		18	6.2		10	3.4		21	7.2	
Fear of infection at medical facilities													
Yes	496	220	44.4	<.001	42	8.5	.004	28	5.6	.953	51	10.3	.067
No	163	29	17.8		3	1.8		9	5.5		9	5.5	
Current medical histories													
Hypertension													
+	324	109	33.6	.031	15	4.6	.028	16	4.9	.458	29	9.0	.892
−	335	140	41.8		30	9.0		21	6.3		31	9.3	
Diabetes													
+	104	34	32.7	.243	7	6.7	.966	4	3.8	.393	9	8.7	.862
−	555	215	38.7		38	6.8		33	5.9		51	9.2	
Heart disease													
+	53	14	26.4	.075	1	1.9	.105	4	7.5	.346	7	13.2	.197
−	606	235	38.8		44	7.3		33	5.4		53	8.7	
Stroke													
+	14	4	28.6	.472	1	7.1	.632	2	14.3	.183	1	7.1	.631
−	645	245	38.0		44	6.8		35	5.4		59	9.1	
Respiratory disease													
+	58	29	50.0	.045	6	10.3	.195	5	8.6	.218	4	6.9	.540
−	601	220	36.6		39	6.5		32	5.3		56	9.3	
Cancer													
+	36	9	25.0	.104	1	2.8	.277	2	5.6	.671	4	11.1	.419
−	623	240	38.5		44	7.1		35	5.6		56	9.0	

^a^ Tokyo suburbs include Saitama, Chiba, and Kanagawa prefecture.

^b^ Northern Kanto includes Gunma, Tochigi, and Ibaraki prefecture.

^c^ Exercise habits are defined as participating light sweating exercise regularly 3 times a week.

* *P*‐value was calculated by chi‐square test.

The rate of “decrease in medical visit frequency” was significantly different by gender (*P* < .001), residential area (*P* = .035), presence or absence fear of being infected at medical facilities (*P* < .001), presence or absence of hypertension (*P* = .031), and respiratory illness (*P* = .045). The rate of “decrease in medical visit frequency” was higher among women, respondents living in the Tokyo Metropolis, respondents with a fear of being infected at medical facilities, respondents without current hypertension, and respondents with respiratory illness.

The rate of “inability to take regular medication” was significantly different with age (*P* = .001), presence or absence of fear of being infected at medical facilities (*P* = .004), and the presence or absence of hypertension (*P* = .028). The rate of “inability to take regular medication” was highest in respondents aged 20‐30 years, respondents who were afraid of being infected at medical facilities, and respondents without hypertension. The rate of “deterioration of chronic illness” was highest in respondents aged 20‐30 years, respondents whose self‐rated health was poor (*P* < .001), and respondents who regularly visited the hospital (*P* = .031). The rate of utilization of telephone/online medical care was significantly higher in participants aged 20‐30 years (*P* = .017) and those who regularly exercised (*P* < .001).

The factors related to “decrease in medical visit frequency” and “inability to take regular medication” are shown in Table 4A. In model 1, females (odds ratio [OR] = 1.73; 95% confidence interval [CI] = 1.16‐2.57; *P* = .007), respondents aged 20‐30 years (OR = 1.93; 95% CI = 1.08‐3.46; *P* = .027), respondents living in the Tokyo Metropolis (OR = 2.12; 95% CI = 1.14‐3.95; *P* = .018), respondents who were married (OR = 1.69; 95% CI = 1.00‐2.86; *P* = .049), respondents whose self‐rated health was poor (OR = 1.71; 95% CI = 1.10‐2.65; *P* = .017), and respondents who were afraid of being infected at medical facilities were significantly more likely to report a “decrease in medical visit frequency.” Even in model 2, which adjusted for the presence or absence of current medical conditions, all covariates except for age were significantly associated with a decreased frequency of visits.

**TABLE 4:** Factors related to treatment‐related behavior/status—Results of multiple logistic regression analyses

(A)	Decrease in medical visit frequency						Inability to take regular medication									
	Model 1^a^			Model 2^b^			Model 1^a^			Model 2^b^			Model 3^c^			
	OR	95%CI	*P*‐value*	OR	95%CI	*P*‐value*	OR	95%CI	*P*‐value*	OR	95%CI	*P*‐value*	OR	95%CI		*P*‐value*
Gender																
Female	1.78	(1.21‐2.61)	.003	1.73	(1.16‐2.57)	.007	0.91	(0.45‐1.85)	.798	0.91	(0.44‐1.88)	.793	0.76	(0.34‐1.67)		.490
Male (ref)	1.00			1.00			1.00			1.00			1.00			
Age (years)																
20‐39	1.93	(1.08‐3.46)	.027	1.61	(0.86‐3.00)	.135	4.80	(1.90‐12.2)	.001	4.03	(1.48‐11.0)	.007	3.49	(1.16‐10.5)		.027
40‐59	1.51	(0.97‐2.34)	.070	1.37	(0.87‐2.17)	.176	2.22	(0.97‐5.10)	.061	1.98	(0.84‐4.68)	.121	1.83	(0.73‐4.59)		.196
60‐79 (ref)	1.00			1.00			1.00			1.00			1.00			
Residential area																
Tokyo Metropolis	2.09	(1.14‐3.86)	.018	2.12	(1.14‐3.95)	.018	0.75	(0.28‐2.03)	.575	0.79	(0.29‐2.16)	.640	0.53	(0.17‐1.66)		.275
Tokyo suburbs	1.50	(0.83‐2.70)	.177	1.45	(0.80‐2.62)	.225	0.63	(0.25‐1.64)	.347	0.62	(0.24‐1.62)	.330	0.50	(0.17‐1.48)		.210
Northern Kanto (ref)	1.00			1.00			1.00			1.00			1.00			
Marital status																
Married	1.66	(0.99‐2.78)	.055	1.69	(1.00‐2.86)	.049	1.49	(0.59‐3.76)	.402	1.56	(0.60‐4.06)	.361	0.97	(0.34‐2.75)		.957
Single, divorced, widowed (ref)	1.00			1.00			1.00			1.00			1.00			
Living arrangement																
Living alone	1.69	(0.91‐3.12)	.095	1.66	(0.89‐3.10)	.109	0.74	(0.22‐2.44)	.615	0.76	(0.22‐2.57)	.657	0.40	(0.11‐1.51)		.179
Living with others (ref)	1.00			1.00			1.00			1.00			1.00			
Education																
University graduate or above	1.02	(0.71‐1.48)	.898	1.03	(0.71‐1.50)	.877	1.06	(0.53‐2.14)	.869	1.11	(0.55‐2.23)	.781	1.09	(0.52‐2.29)		.821
Less than junior college graduate (ref)	1.00			1.00			1.00			1.00			1.00			
Working status																
Yes	1.03	(0.63‐1.69)	.895	1.00	(0.61‐1.64)	.992	1.90	(0.85‐4.22)	.116	1.83	(0.82‐4.08)	.138	1.28	(0.56‐2.94)		.555
No (ref)	1.00			1.00			1.00			1.00			1.00			
Annual household income																
Less than 5 million JPY	0.72	(0.50‐1.04)	.080	0.72	(0.49‐1.05)	.084	1.08	(0.53‐2.22)	.830	1.09	(0.53‐2.23)	.825	1.33	(0.60‐2.93)		.486
More than 5 million JPY (ref)	1.00			1.00			1.00			1.00			1.00			
Smoking status																
Smoker	1.28	(0.89‐1.84)	.181	1.27	(0.88‐1.84)	.196	1.11	(0.56‐2.18)	.763	1.13	(0.57‐2.23)	.731	1.83	(0.72‐4.63)		.203
Nonsmoker or ex‐smoker (ref)	1.00			1.00			1.00			1.00			1.00			
Alcohol consumption																
More than 3days / week	1.17	(0.82‐1.68)	.378	1.18	(0.82‐1.70)	.363	0.92	(0.47‐1.80)	.806	0.92	(0.46‐1.82)	.803	0.90	(0.43‐1.90)		.788
Less than 3days / week (ref)	1.00			1.00			1.00			1.00			1.00			
Exercise habits																
Yes	0.96	(0.64‐1.44)	.847	0.95	(0.64‐1.43)	.822	1.20	(0.56‐2.59)	.639	1.19	(0.54‐2.62)	.668	0.70	(0.33‐1.50)		.366
No (ref)	1.00			1.00			1.00			1.00			1.00			
Self‐rated health																
Not very well, not good	1.62	(1.06‐2.48)	.027	1.71	(1.10‐2.65)	.017	0.93	(0.40‐2.15)	.862	0.96	(0.41‐2.26)	.929	0.59	(0.23‐1.54)		.280
Good, very good (ref)	1.00			1.00			1.00			1.00			1.00			
Type of medical facilities participants were visiting																
Hospital	0.75	(0.53‐1.05)	.091	0.80	(0.57‐1.14)	.213	1.10	(0.58‐2.10)	.768	1.20	(0.62‐2.33)	.581	1.42	(0.7‐2.9)		.332
Clinic (ref)	1.00			1.00			1.00			1.00			1.00			
Fear of infection at medical facilities																
Yes	4.00	(2.52‐6.36)	<.001	3.92	(2.46‐6.25)	<.001	6.23	(1.85‐20.9)	.003	6.16	(1.83‐20.8)	.003	3.39	(0.89‐12.84)		.073
No (ref)	1.00			1.00			1.00			1.00			1.00			
Current medical histories																
Hypertension																
+				0.84	(0.58‐1.22)	.365				0.69	(0.33‐1.43)	.316	0.73	(0.33‐1.61)		.432
− (ref)				1.00						1.00			1.00			
Diabetes																
+				0.98	(0.60‐1.63)	.948				1.45	(0.57‐3.70)	.432	1.75	(0.61‐5.01)		.297
− (ref)				1.00						1.00			1.00			
Heart disease																
+				0.53	(0.26‐1.07)	.075				0.27	(0.03‐2.14)	.214	0.40	(0.05‐3.57)		.413
− (ref)				1.00						1.00			1.00			
Stroke																
+				0.88	(0.26‐3.02)	.841				1.32	(0.15‐11.2)	.802	1.99	(0.19‐21.11)		.569
− (ref)				1.00						1.00			1.00			
Respiratory disease																
+				1.46	(0.81‐2.65)	.212				1.60	(0.60‐4.26)	.349	1.59	(0.55‐4.63)		.393
− (ref)				1.00						1.00			1.00			
Cancer																
+				0.51	(0.22‐1.19)	.121				0.52	(0.07‐4.10)	.534	0.79	(0.09‐6.96)		.831
− (ref)				1.00						1.00			1.00			
Decrease in medical visit frequency																
Yes													28.5	(8.31‐97.56)	<.001	
No (ref)													1.00			

(B)	Deterioration of chronic disease										Utilization of telephone/online medical care					
	Model 1^a^				Model 2^b^			Model 3^c^			Model 1^a^			Model 2^b^		
	OR		95%CI	p‐value*	OR	95%CI	*P*‐value*	OR	95%CI	*P*‐value*	OR	95%CI	*P*‐value*	OR	95%CI	*P*‐value*
Gender																
Female	1.59	(0.72‐3.54)		.255	1.68	(0.73‐3.87)	.225	1.57	(0.67‐3.67)	.298	1.26	(0.68‐2.36)	.464	1.28	(0.67‐2.47)	.459
Male (ref)	1.00				1.00			1.00			1.00			1.00		
Age (years)																
20–39	5.79		(1.99‐16.8)	.001	7.18	(2.21‐23.3)	.001	6.92	(2.12‐22.6)	.001	1.75	(0.74‐4.15)	.203	1.83	(0.72‐4.69)	.206
40–59	2.85		(1.08‐7.57)	.035	3.00	(1.10‐8.22)	.032	2.86	(1.04‐7.87)	.042	0.45	(0.18‐1.11)	.082	0.45	(0.18‐1.14)	.093
60–79 (ref)	1.00				1.00			1.00			1.00			1.00		
Residential area																
Tokyo Metropolis	4.41		(0.54‐36.3)	.167	4.93	(0.58‐41.6)	.143	4.00	(0.47‐33.8)	.203	2.00	(0.71‐5.69)	.192	1.99	(0.69‐5.68)	.201
Tokyo suburbs	3.91		(0.49‐31.2)	.198	4.53	(0.55‐37.2)	.159	3.97	(0.48‐32.6)	.199	1.28	(0.46‐3.57)	.635	1.28	(0.46‐3.60)	.639
Northern Kanto (ref)	1.00				1.00			1.00			1.00			1.00		
Marital status																
Married	1.41		(0.52‐3.84)	.506	1.37	(0.50‐3.79)	.540	1.31	(0.47‐3.64)	.610	1.49	(0.59‐3.72)	.399	1.45	(0.58‐3.61)	.429
Single, divorced, widowed (ref)	1.00				1.00			1.00			1.00			1.00		
Living arrangement																
Living alone	1.40		(0.42‐4.59)	.584	1.36	(0.41‐4.59)	.617	1.33	(0.39‐4.48)	.651	1.91	(0.69‐5.29)	.216	1.90	(0.68‐5.29)	.223
Living with others (ref)	1.00				1.00			1.00			1.00			1.00		
Education																
University graduate or above	0.66		(0.30‐1.47)	.313	0.67	(0.30‐1.52)	.340	0.62	(0.27‐1.43)	.266	1.01	(0.55‐1.85)	.984	0.99	(0.54‐1.84)	.979
Less than junior college graduate (ref)	1.00				1.00			1.00			1.00			1.00		
Working status																
Yes	1.95		(0.78‐4.88)	.152	2.14	(0.84‐5.44)	.109	1.61	(0.63‐4.09)	.318	0.70	(0.29‐1.70)	.431	0.71	(0.29‐1.72)	.445
No (ref)	1.00				1.00			1.00			1.00			1.00		
Annual household income																
Less than 5 million JPY	0.69		(0.30‐1.60)	.386	0.72	(0.31‐1.70)	.452	0.76	(0.32‐1.79)	.526	0.95	(0.51‐1.74)	.859	0.92	(0.50‐1.70)	.789
More than 5 million JPY (ref)	1.00				1.00			1.00			1.00			1.00		
Smoking status																
Smoker	0.58		(0.24‐1.39)	.217	0.58	(0.24‐1.40)	.223	2.26	(0.87‐5.85)	.093	1.34	(0.75‐2.41)	.322	1.36	(0.75‐2.45)	.308
Nonsmoker or ex‐smoker (ref)	1.00				1.00			1.00			1.00			1.00		
Alcohol consumption																
More than 3 d/wk	1.53		(0.69‐3.43)	.298	1.59	(0.70‐3.61)	.264	0.55	(0.22‐1.35)	.190	2.60	(1.47‐4.62)	.001	2.64	(1.47‐4.72)	.001
Less than 3 d/wk (ref)	1.00				1.00			1.00			1.00			1.00		
Exercise habits																
Yes	1.54		(0.63‐3.78)	.346	1.64	(0.65‐4.10)	.294	1.43	(0.62‐3.28)	.401	0.77	(0.40‐1.49)	.431	0.76	(0.39‐1.49)	.423
No (ref)	1.00				1.00			1.00			1.00			1.00		
Self‐rated health																
Not very well, not good	6.21		(2.86‐13.5)	< .001	5.71	(2.57‐12.7)	<.001	5.19	(2.31‐11.7)	<.001	0.89	(0.42‐1.88)	.764	0.88	(0.41‐1.88)	.736
Good, very good (ref)	1.00				1.00			1.00			1.00			1.00		
Type of medical facilities participants were visiting																
Hospital	1.62		(0.74‐3.58)	.230	1.52	(0.67‐3.42)	.317	1.62	(0.71‐3.68)	.249	1.82	(1.02‐3.24)	.043	1.79	(0.99‐3.23)	.054
Clinic (ref)	1.00				1.00			1.00			1.00			1.00		
Fear of infection at medical facilities																
Yes	1.51		(0.64‐3.55)	.349	1.56	(0.64‐3.78)	.327	1.16	(0.46‐2.93)	.755	1.77	(0.82‐3.80)	.144	1.77	(0.82‐3.79)	.146
No (ref)	1.00				1.00			1.00			1.00			1.00		
Current medical histories																
Hypertension																
+					1.55	(0.67‐3.56)	.304	1.63	(0.71‐3.75)	.253				0.99	(0.53‐1.83)	.961
− (ref)					1.00			1.00						1.00		
Diabetes																
+					0.72	(0.20‐2.61)	.621	0.72	(0.20‐2.62)	.615				0.99	(0.44‐2.23)	.975
− (ref)					1.00			1.00						1.00		
Heart disease																
+					1.60	(0.44‐5.73)	.474	1.88	(0.53‐6.74)	.332				1.49	(0.60‐3.71)	.389
− (ref)					1.00			1.00						1.00		
Stroke																
+					2.15	(0.32‐15.0)	.430	2.04	(0.30‐13.7)	.465				1.04	(0.12‐9.07)	.970
− (ref)					1.00			1.00						1.00		
Respiratory disease																
+					1.51	(0.50‐4.58)	.464	1.37	(0.44‐4.26)	.591				0.61	(0.20‐1.88)	.390
− (ref)					1.00			1.00						1.00		
Cancer																
+					1.45	(0.28‐7.49)	.654	1.59	(0.30‐8.33)	.586				1.07	(0.33‐3.44)	.914
− (ref)					1.00			1.00						1.00		
Decrease in medical visit frequency																
Yes								2.26	(1.01‐5.04)	.047						
No (ref)								1.00								

^a^ Independent variables of model 1 were gender, age, residential area, marital status, living arrangement, education, working status, annual household income, lifestyle (smoking status, alcohol consumption, and exercise habits), self‐reported health, type of medical facility participants were visiting, and fear of being infected at medical facilities.

^b^ In model 2, in addition to the independent variables discussed in model 1, the presence or absence of a current illness (hypertension, diabetes, heart disease, stroke, respiratory disease, and cancer) was input as an independent variables.

^c^ In model 3, in addition to the independent variables discussed in model 2, decrease in visit frequency was input as an independent variable.

* *P*‐value was calculated by a multivariable logistic regression analysis.

Respondents aged 20‐30 years (OR = 4.03; 95% CI = 1.48‐11.0; *P* = .007) and who were afraid of being infected at medical facilities (OR = 6.16; 95% CI = 1.83‐20.8; *P* = .003) were significantly more likely to report an “inability to take regular medication.”

Respondents aged 20‐30 years (OR = 7.18; 95% CI = 2.21‐23.3; *P* = .001), those aged 40‐50 years (OR = 3.00; 95% CI = 1.10‐8.22; *P* = .032), and those whose self‐rated health was poor (OR = 5.71; 95% CI = 2.57‐12.67; *P* < .001) were significantly more likely to report a “deterioration of chronic disease” (Table 4B).

In the results of model 3, "inability to take regular medication" and "worsening of chronic disease" were significantly associated with "decrease in medical visit frequency" (OR = 28.5; 95% CI = 8.31‐97.56; *P* < .001, OR = 2.26; 95% CI = 1.01‐5.04; *P* = .047, respectively) (Table 4A,B).

Respondents whose visiting medical institution was a hospital (OR = 1.79; 95% CI = 0.99‐3.23; *P* = .054) had a nonsignificantly higher tendency to use telephone/online medical care than those who had visited a clinic.

## 4 DISCUSSION

Among patients who had regularly scheduled outpatient visits, the proportion of patients who visited less frequently during the first wave of COVID‐19 was high—approximately 40% in the Tokyo metropolitan area. However, fewer than 10% of patients were unable to take their regular medications or reported deterioration in their chronic illnesses. Fear of being infected at medical facilities was strongly associated with a decrease in both visit frequency and an inability to take regular medications.

In this study, which was conducted in May 2020 just before the state of emergency was lifted in all prefectures for the first wave of COVID‐19, we found that the frequency of visits had decreased by 37.8%. We believe that our study is the first to report the degree of decrease in the frequency of medical treatment for those who regularly visit medical facilities. On the basis of the number of billing receipts, the Japanese Ministry of Health, Labor and Welfare (Central Social Insurance Medical Council) reported that the number of outpatient clinic visits in May 2020 was 79% of the previous year.^10^ Although it is not possible to directly compare the results of these two studies, we considered the following reasons for the discrepancy between the percentages. First, it is possible that the decrease in the frequency of visits in this study can be explained by the inclusion of visits in departments other than the internal medicine department; nationally, decreases in receipt counts have been reported for specific departments such as pediatrics or otolaryngology.^10^ Second, those who received telephone/online medical care did not go directly to the medical facilities; therefore, they may have reported that their “frequency of visits has decreased.”

Overall, the decrease in medical visit frequency was relatively high at approximately 40%. Furthermore, only about 7% of patients were unable to take regular medications, and only 5.6% reported a deterioration of their illness. Following a Ministry notification^15^ on February 28 recommending long‐term prescriptions, many doctors may have prevented patients from running out of regular medications by giving long‐term prescriptions. According to an analysis of the Japan Medical Information Research Institute's prescription information database, the average number of prescription days in April and May increased by 18% and 16%, respectively, from the same month of the previous year.^16^ In each month, the average number of prescription days increased compared with that of the previous year. In general, it cannot be said whether more frequent visits to medical institutions are better, and a decrease in the number of visits is not necessarily judged to be problematic. Particularly, during the COVID‐19 pandemic, it is also important to consider the appropriate use of medical human resources. Adjustment of the medical visit interval between visits as well as utilizing telephone/online visits should be considered for chronic diseases according to the severity of the disease.

The factors associated with a decrease in medical visit frequency during the early wave of COVID‐19 were fear of being infected at medical facilities, residency in the Tokyo Metropolis, self‐rated poor health, and being married. One reason why the fear of being infected at medical facilities was strongly related to the decrease in visit frequency may be that a large cluster of COVID‐19 patients in the inpatient ward of a hospital in Tokyo was widely reported in the news.^17^ Gender was also significantly associated with a decrease in medical visit frequency. Women were 1.7 times more likely to have fewer medical visits than men. A previous study reported that there was only a slight gender difference in the decrease in visit frequency for patients with medical needs (the rate of decrease in medical visit frequency was 34% for men and 37% for women).^18^ Various factors may be playing an intertwined role in medical visit frequency. Age (a younger age is correlated with fewer medical visits), economic situation (a younger age and financial distress are correlated with fewer consultations), and time constraints may all affect visit frequency. Given that this study was conducted under the special circumstances of the first wave of COVID‐19, we cannot be sure of the role that COVID‐19 played for women during this period compared with the periods used in previous studies. Women may have been more likely to reduce their medical visit frequency because, at almost all ages, they were more likely than men to fear being infected at medical facilities. We have previously reported that women were significantly more likely than men to implement the five WHO recommendations to prevent COVID‐19 infection.^19^ Women were also more aware of their hygienic environment and more concerned about infection, thus possibly affecting the frequency of visits. The lower frequency of medical visits and failure to take medication regularly, especially among younger respondents, may have been influenced by the lower severity of their underlying diseases. The finding that living in the Tokyo Metropolis was related to the decrease in visiting frequency is plausible as Tokyo has the country's highest population density and the highest rate of infections.

In previous studies, the elderly and patients with underlying diseases, notably hypertension, diabetes, cardiovascular disease, respiratory disease, and malignant tumors, are at high risk of becoming very ill when infected with COVID‐19.^4^, ^5^, ^6^, ^7^, ^8^, ^9^ In Japan, it was feared that medical visits of the elderly and patients undergoing regular medical treatments for chronic diseases would be greatly affected. However, in our study, elderly respondents (aged 60‐70 years) did not report significant decreases in medical visit frequency, an inability to take regular medications, or a deterioration of chronic illness. Young people aged 20‐30 years were more likely to report an inability to take regular medications. Decrease in medical visit frequency, inability to take regular medication, and deterioration of chronic illness were not associated with any specific diseases.

This study found that patients undergoing regular outpatient visits were “afraid of being infected with COVID‐19 at medical facilities,” and that this was strongly associated with a decrease in medical visit frequency and an inability to take regular medications. To reduce the number of patients who drop out of regular medical visits owing to a fear of being exposed to COVID‐19, it is important to continue implementing thorough infection control measures at medical facilities and to inform patients about these efforts. On the other hand, in the current situation where physical distancing is required, it is also necessary to develop and encourage the use of indirect visiting methods such as telephone and online medical care. Indirect outpatient visits may improve patients’ access to their regular medications and thus prevent deterioration of their chronic illnesses. Although online medical care is not widely used in Japan, international data indicate that the number of patient visits to fever clinics has decreased by 50% since the start of online medical care.^20^ Introducing online medical care poses a financial burden to provider organizations, but telephone medical care only requires a small investment in new equipment. To prevent the decrease in medical visit frequency and inability to access regular medications for patients who are afraid of being infected at medical facilities, the availability of telephone medical care should be increased prior to the next wave of COVID‐19. The results of this study showed that the hospital‐visiting groups tended to utilize telephone medical care more than the clinic‐visiting groups. There is an opportunity for more telephone medical care in clinics, but it does not allow for physical examinations or bloodwork; therefore, it is necessary to consider further measures to prevent the deterioration of chronic diseases. In the past, doctors were required to be trained before providing online medical care, in accordance with the JMHLW “Guidelines for Proper Implementation of Online Medical Care”.^21^ However, considering the spread of COVID‐19 in April, even doctors who are not currently taking the course can provide telephone/online medical care. It would be useful to inform medical facilities of this training and encourage doctors to undergo learning opportunities regarding the provision of online medical care.^22^

The limitations of our study are as follows. First, participants in this study were recruited via an Internet research company and the survey was conducted via email. This may have resulted in selection bias, as the target population was based on people with relatively high IT literacy. Fewer than 10% of respondents reported utilizing telephone or online care; the actual utilization in the general population may be even lower. Furthermore, in this study, “telephone medical care” and “online medical care” were collectively investigated as “telephone/online medical care.” Therefore, it is not possible to discuss the utilization status of telephone medical care and online medical care separately. At the time of the study, online medical care was not widely used in Japan and most of the answers likely reflected telephone medical care.

Second, the participants’ ages were in the 20s to 70s. Although it has been indicated that advanced age is a risk factor for COVID‐19 aggravation, this study could not clarify the outpatient status of elderly people >80 years old.

Third, the evaluation of the deterioration of a chronic disease is self‐reported and may lack objectivity. In addition, this study may have been conducted too early in the course of COVID‐19 to accurately assess the degree of deterioration. Finally, this study was conducted during the first wave of COVID‐19 in Japan. If this study was to be conducted during subsequent waves of COVID‐19, it is unclear whether the results would be the same. It is also unclear whether the results of this study can be applied to patients in different countries.

Despite the above limitations, this study clarified the medical treatment status and related factors of outpatients at risk of COVID‐19 aggravation during the first wave of COVID‐19. As of fall 2020, COVID‐19 has not disappeared. As the pandemic continues, medical facilities will need to thoroughly prevent infections and promote telephone/online medical care to mitigate the decrease in in‐person visits, and the inability to take regular medications.

## 5 CONCLUSION

This cross‐sectional study found that the frequency of outpatient visits decreased approximately 40% during the first wave of COVID‐19, but that fewer than 10% of the respondents had run out of their regular medications or had a deterioration of a chronic disease. Fear of being infected with COVID‐19 at medical facilities was strongly associated with a decrease in medical visit frequency and inability to take regular medications. It is important to continue implementing thorough infection control measures at medical facilities and to inform the public the importance of keeping chronic diseases in good condition as well as promoting telephone/online medical care to avoid decreases in medical visit frequency.

## CONFLICT OF INTEREST

The authors have stated explicitly that there are no conflicts of interest in connection with this article.

## AUTHOR CONTRIBUTIONS

TTakakubo, YO, and SI designed the study. MM and SI participated in the data collection. TTakakubo and YO undertook the statistical analysis and wrote the manuscript. TTakamiya, NF, HK, SA, IN, and HW assisted in the revision of the manuscript. All authors contributed to and approved the final manuscript.

## ETHICAL APPROVAL

This study was approved by the Ethics Committee of Tokyo Medical University, Tokyo, Japan (No: T2019‐0234). Informed consent was obtained from all respondents.
